# A first-in-class POLRMT specific inhibitor IMT1 suppresses endometrial carcinoma cell growth

**DOI:** 10.1038/s41419-023-05682-7

**Published:** 2023-02-23

**Authors:** Shu-ping Li, Li Ou, Yan Zhang, Fang-rong Shen, You-guo Chen

**Affiliations:** 1 Department of Obstetrics and Gynecology, The First Affiliated Hospital of Soochow University, Suzhou, China; 2 Obstetrics Department, The Affiliated Changzhou No.2 People’s Hospital of Nanjing Medical University, Changzhou, China; 3 Department of Obstetrics and Gynecology, The Second Affiliated Hospital of Soochow University, Suzhou, China; 4 Department of Radiotherapy and Oncology, Affiliated Kunshan Hospital of Jiangsu University, Kunshan, China

**Keywords:** Endometrial cancer, Targeted therapies

## Abstract

Exploring novel molecularly-targeted therapies for endometrial carcinoma is important. The current study explored the potential anti-endometrial carcinoma activity by a first-in-class POLRMT (RNA polymerase mitochondrial) inhibitor IMT1. In patient-derived primary human endometrial carcinoma cells and established lines, treatment with IMT1 potently inhibited cell viability, proliferation, cell-cycle progression and motility, while inducing robust caspase-apoptosis activation. Treatment with the PLORMT inhibitor impaired mitochondrial functions, leading to mtDNA (mitochondrial DNA) transcription inhibition, mitochondrial membrane potential decline, reactive oxygen species formation, oxidative stress and ATP loss in the endometrial carcinoma cells. Similarly, POLRMT depletion, through shRNA-induced silencing or CRISPR/Cas9-caused knockout (KO), inhibited primary endometrial carcinoma cell proliferation and motility, and induced mitochondrial dysfunction and apoptosis. Importantly, IMT1 failed to induce further cytotoxicity in POLRMT-KO endometrial carcinoma cells. Contrarily, ectopic overexpression of POLRMT further augmented proliferation and motility of primary endometrial carcinoma cells. In vivo, oral administration of a single dose of IMT1 substantially inhibited endometrial carcinoma xenograft growth in the nude mice. mtDNA transcription inhibition, oxidative stress, ATP loss and apoptosis were detected in IMT1-treated endometrial carcinoma xenograft tissues. Together, targeting PLORMT by IMT1 inhibited endometrial carcinoma cell growth in vitro and in vivo.

## Introduction

Endometrial carcinoma is one common gynecological cancer causing huge economic/social burden and large cancer-related human mortalities globally [1, 2]. Its incidence is still rising in major developing and developed countries [3–5]. An ageing population, few benign hysterectomies and obesity are the primary causes of this trend [3–5]. Early-stage endometrial carcinomas with presentation of postmenopausal bleeding could be cured by hysterectomy [1, 2]. Yet for those with advanced diseases, including recurrent, metastatic and therapy-resistant endometrial carcinomas, the prognosis is often poor [1, 2]. Early diagnosis and effective treatments for this devastating disease are still challenging [1, 2]. Further exploring the pathological mechanisms of endometrial carcinoma progression should advance targeted chemotherapeutic strategies [3–5].

To meet the increased energy demand, cancer cells have enhanced glycolysis when compared to the normal cells [6, 7]. Intriguingly, recent findings have suggested that oxidative phosphorylation (OXPHOS) could also be augmented in certain human cancers, including leukemia, lymphoma, pancreatic cancer, and endometrial carcinoma [6–9]. It could even occur in cancer cells with active glycolysis [6–9]. Inhibition of OXPHOS, by pharmacologic inhibitors or genetic means, have shown promising potential in inhibiting the growth of cancer cells with upregulated OXPHOS [6–9].

POLRMT, or RNA polymerase mitochondrial, is one primary component of the mitochondrial transcription machinery and is essential for OXPHOS and mitochondrial biogenesis [10, 11]. POLRMT, along with mitochondrial transcription factor A (TFAM) and B2 (TFB2M), are responsible for the initiating and progressing of the transcription of mitochondrial DNA (mtDNA) [10, 11]. Furthermore, it is also vital for the synthesis of RNA primers, required for mtDNA replication [12, 13]. A few recent studies have proposed the important cancer-promoting action by POLRMT. POLRMT silencing largely inhibited OXPHOS and hindered acute myeloid leukemia (AML) cell growth [14]. A POLRMT inhibitor, 2-C-methyladenosine, potently suppressed AML cell growth in vitro and in vivo [14]. In breast cancer cells, elevated POLRMT expression and mitochondrial biogenesis (through OXPHOS) promoted cancer cell growth [15, 16]. Bonekamp et al., have recently developed IMT1 as a first-in-class specific and noncompetitive POLRMT inhibitor [17, 18]. It was able to block mitochondrial transcription, and disrupted OXPHOS and mitochondrial biogenesis [17]. The current study explored the potential anti-endometrial carcinoma activity by IMT1 and the possible underlying mechanisms.

## Materials and methods

### Reagents

Polybrene, n-acetylcysteine (NAC), ATP, z-DEVD-fmk, z-VAD-fmk, and puromycin were provided by Sigma-Aldrich (St. Louis, MO). IMT1 and antibodies were from Dr. Wang [19]. Ataxia telangiectasia mutated kinase (ATM) and ataxia telangiectasia and Rad3-related kinase (ATR) antibodies were provided by Dr. Xu [20]. Fluorescence probes, including TUNEL, tetraethylbenzimidazolylcarbocyanine iodide (JC*-*1), DAPI (4′,6-diamidino-2-phenylindole), CellROX, dichlorodihydrofluorescein diacetate (DCF-DA), propidium iodide (PI) were obtained from Thermo-Fisher Invitrogen (Shanghai, China).

### Cell culture

At the time of tumor resection surgery, the fresh endometrial carcinoma tissues and adjacent normal endometrial epithelial tissues, derived from different primary patients, were carefully separated under microscopy. Fresh human tissues were then cut into small pieces and were digested through collagenase and dispase II. After four hours, the digested cells were washed, centrifuged, and filtered. The primary cancer cells or the primary epithelial cells were cultivated in high glucose DMEM/F-12 growth medium with 12% FBS, 0.5 μg/mL hydrocortisone, 2.5 μg/mL insulin, 7.5 ng/mL epidermal growth factor (EGF), 10 μg/mL adenine and necessary antibiotics. The primary cancer cells that were derived from the three patients were named as “phEC-1”, “phEC-2” and “phEC-3”. The primary endometrial epithelial cells that were derived from two patients were named as “phEE-1” and “phEE-2”. The immortalized endometrial carcinoma cell lines, HEC-1 and KLE, were purchased from the Cell Bank of Shanghai Institute of Biological Sciences (Shanghai, China) and cells were maintained in DMEM/RPMI medium with 10% FBS. Each patient provided written-informed consent. The protocols of using primary human cells were approved by Ethics committee of Soochow University.

### EdU (5-ethynyl-20-deoxyuridine) incorporation

Cells were seeded at 70–80% confluence. EdU Apollo-567 In Vitro Imaging Kit (Ribo-Bio, Guangzhou, China) was employed. In brief, after the described treatments, EdU (15 μM) was added to stain the cell nuclei, which were co-stained with DAPI. After several washes with PBS, the cell nuclei were visualized by a fluorescent microscope (Leica).

### Colony formation

In brief, cells were first seeded into 10-cm culturing dish at 20,000 cells per well and maintained in complete medium. Medium was renewed every 2–3 days. After 14 days cell colonies were fixed, stained and manually counted.

### Cell migration

“Transwell” chambers (8-µm pore size, Corning) were utilized to test in vitro cell migration. Brief, cells (20,000 cells per chamber) were re-suspended in basic medium without serum and coated on the upper surface of chambers. To the lower compartment, 600 µL serum (10%)-containing complete medium was added. After16 h, cells remained on the upper surfaces were gently scraped. Cells that migrated to the lower chamber membranes were fixed and stained.

### Caspase activity

The caspase-3/-7 activities were measured by a commercial kit (Abcam, Shanghai, China). Briefly, cells were seeded into 96-well plates at 3.5 × 10^3^ cells per well and subject to the applied treatments. After 48 h, cell lysates were incubated with the loading solution including the caspase-3/-7 substrate for 45 min. Then, caspase-3/-7 activities were measured through a fluorescence microplate reader (BioTek Synergy), and 625 nm emission for caspase-3 and 455 nm emission for caspase-7.

### Apoptosis assays

The protocols of nuclear TUNEL staining assay were described in other studies [21–23].

### POLRMT shRNA or overexpression

The POLRMT shRNA lentivirus or PLORMT cDNA construct-expressing lentivirus were provided by Dr. Shi at Soochow University [24]. Endometrial carcinoma cells were seeded at 55-65% confluence in polybrene-containing complete medium and were infected with the lentivirus at MOI = 12. Viral infection was repeated after 24 h. After another 48 h, cells were maintained in fresh medium containing puromycin (4 μg/mL). Stable cells, with the POLRMT shRNA or the PLORMT overexpression construct, were then established after another 48 h. Control cells were infected with lentivirus-packed scramble control shRNA or lentivirus-packed empty vector. Expression of PLORMT in the cells was always checked by qRT-PCR and Western blotting assays.

### CRSIPR/Cas9-induced POLRMT knockout (KO)

First, endometrial carcinoma cells were seeded at 55–65% confluence in polybrene-containing medium and were infected with the lentivirus encoding the Cas9-expressing construct. Puromycin was thereafter added to select the stable Cas9-expressing cells. Next, the CRSIPR/Cas9-POLRMT-KO-construct, encoding sgRNA against human *POLRMT* (also from Dr. Shi [24]), was transduced to Cas9-expressing cells. Thereafter, the transfected cells were distributed into 192-well plates. These cells in each well were subject to POLRMT KO screening and POLRMT KO colonies were then formed. Control cells were stably transduced with the CRSIPR/Cas9-KO control construct.

### ROS (reactive oxygen species) assays

Cells with applied treatments were stained with DCF-DA or CellROX. After extensive washes, DCF-DA or CellROX fluorescence were visualized by a fluorescence microscopy (Leica) and images were taken. To quantify fluorescence intensity, cells were measured under a fluorescence spectrophotometer (F-7000, Hitachi-Hightech).

### Single strand DNA (ssDNA) ELISA

The lysates of the cells with the described treatments were measured under a ssDNA ELISA kit (Invitrogen), and ELISA OD (indicating ssDNA contents) examined at 450 nm in each well.

### ATP contents and the mitochondrial complex I activity

The lysates of the cells with the described treatments were measured by an ATP assay kit (Biyuntian, Wuxi, China) and a mitochondrial complex I activity kit (Biyuntian, Wuxi, China). The cellular ATP levels and mitochondrial complex I activity were then tested based the attached protocols.

### Thiobarbituric acid reactive substance (TBAR) assay

Lipid peroxidation intensity (25 μg lysates per treatment) was tested through a TBAR kit (Cayman Chemical, MI) according to the attached protocols. TBAR reaction reagent colorimetrically quantified lipid peroxidation and quantified malondialdehyde (MDA) intensity. TBAR intensity was measured at 535 nm of the reference wavelength [25]. Its value, in nmol per mg of total protein, was normalized to that of control.

### Other assays

including Western blotting, qRT-PCR, mitochondrial depolarization detection by JC-1 staining and CCK-8 cell viabilty, as well as Trypan blue staining of cell death intensity and [H^3^] DNA incorporation ELISA were reported in detail in previous studies [26–30]. Figure S1 summarized uncropped blotting images of the study.

### Xenograft studies

The phEC-1 primary cancer cells (at 6 × 10 ^6^ cells per mouse, in 0.2 mL FBS-free DMEM/Matrigel medium) were injected subcutaneously (s.c.) to the flanks of the nude mice (described previously [19]). The subcutaneous phEC-1 xenografts were formed after 18 days and each xenograft was close to 100 mm^3^ in the volume. The xenograft-bearing mice were then orally administrated with IMT1 or vehicle control [17]. IMT1 was administrated at 50 mg/kg body weight, every 48 h for five rounds (at day-0, day-2, day-4, day-6 and day-8). The animal body weights and xenograft volumes were tested every six days using the described method [31]. Animal protocols have been approved by IACUC and Ethics Committee of Soochow University.

### Statistical analysis

In vitro experiments were repeated five times. Data were always with normal distribution and were presented as mean ± standard deviation (SD). Statistical analyses were carried out through the SPSS 23.0 software (SPSS Co., Chicago, IL). Unpaired student’s T-test was employed to compare two groups. One-way ANOVA with the Scheffe’ and Tukey Test was employed for comparison of more than two groups. *P* values of <0.05 were considered statistically significant.

## Results

### IMT1 exerts significant anti-cancer activity in cultured endometrial carcinoma cells

**Fig. 1:**
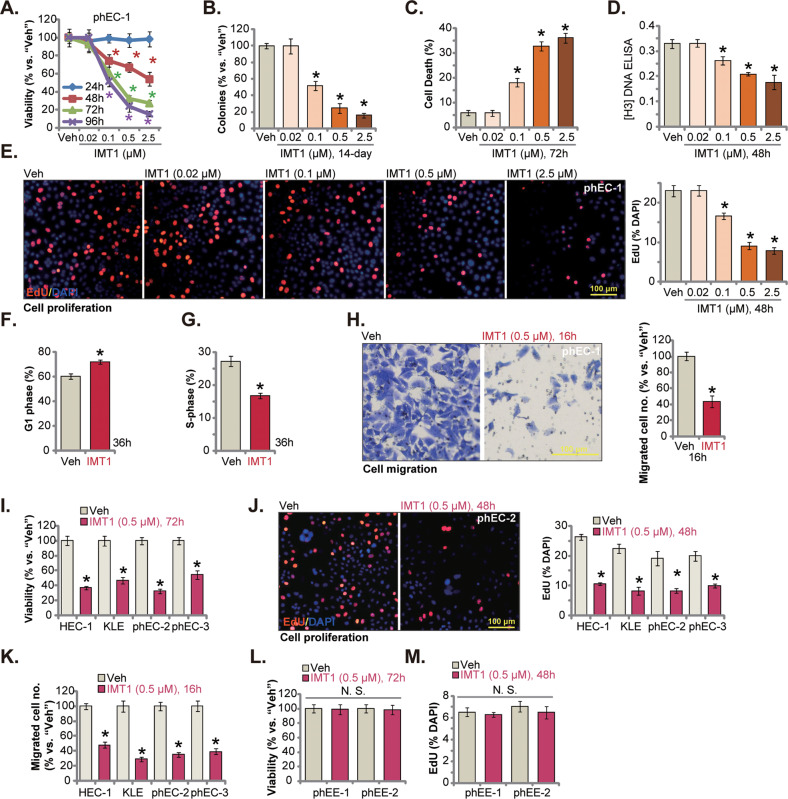
IMT1 exerts significant anti-cancer activity in cultured endometrial carcinoma cells. The primary human endometrial carcinoma cells, phEC-1, were treated with IMT1 at the designated concentration and further cultivated for designated time, cell viability (**A**), colony formation (**B**), cell death (by recording Trypan blue-positive cell ratio, **C**), [H^3^] DNA intensity (**D**) and EdU incorporation (**E**) as well as quantified cell cycle percentages (PI-FACS assays, **F** and **G**) and in vitro cell migration (**H**) were measured by the corresponding assays. The immortalized endometrial carcinoma cell lines (HEC-1/KLE), primary cancer cells (“phEC-2/phEC-3”) or the human endometrial epithelial cells (“phEE-1/-2”) were treated with IMT1 (0.5 μM), cells were further cultivated for designated hours, cell viability (**I** and **L**), proliferation (by measuring nuclear EdU incorporation, **J** and **M**) and in vitro cell migration (**K**) were tested similarly. “Veh” stands for the vehicle control (0.1% DMSO). Data were presented as mean ± standard deviation (SD, *n* = 5). *P < 0.05 vs. “Veh” treatment. “N.S.” stands for the non-statistical difference (*P* > 0.05). The experiments were repeated five times with similar results obtained. Scale bar = 100 μm.

First, the primary human endometrial carcinoma cells, phEC-1 (see “Methods”), were cultivated under FBS-containing complete medium and were treated with different concentrations of IMT1 (0.02/0.1/0.5/2.5 μM). The CCK-8 assay results, Fig. 1A, revealed that IMT1 efficiently decreased the viability of phEC-1 cells. IMT1 displayed a concentration-dependent activity in inhibiting phEC-1 cell survival and was significant at 0.1/0.5/2.5 μM (Fig. 1A). A time-dependent response was noticed as well and the POLRMT inhibitor required at least 48 h to cause significant viability reduction (Fig. 1A). IMT1, at 0.1/0.5/2.5 μM, substantially decreased the number of viable phEC-1 cell colonies (Fig. 1B). In addition, the POLRMT inhibitor dose-dependently increased the number of Trypan blue-positive cells, causing significant cell death (Fig. 1C). Furthermore, IMT1 robustly inhibited phEC-1 cell proliferation and inhibited [H^3^] DNA incorporation (Fig. 1D). To further support the anti-proliferative activity of IMT1, we showed that EdU-positive nuclei ratio was robustly decreased following 0.1/0.5/2.5 μM of IMT1 treatment in phEC-1 cells (Fig. 1E). These titration experiments in Fig. 1A–E found that 0.5 μM of IMT1 resulted in robust anti-survival, cytotoxic and anti-proliferative activity in phEC-1 cells. This concentration was close to IC-50 and was selected for following in vitro experiments.

Further PI flow cytometry assay results found that treatment with 0.5 μM of IMT1 inhibited phEC-1 cell cycle progression (Fig. 1F, G). It increased G1-phase cells’ percentage and reduced S-phase cells’ percentage, leading to G1-S cell cycle arrest (Fig. 1F, G). Moreover, treatment with the POLRMT inhibitor at 0.5 μM potently inhibited phEC-1 cell in vitro migration (Fig. 1H), which was measured by “Transwell” assays.

The potential activity of IMT1 in other endometrial carcinoma cells was explored. The immortalized endometrial carcinoma cell lines (HEC-1/KLE) and primary cancer cells (“phEC-2/phEC-3”, -derived from two other patients) were treated with 0.5 μM of IMT1. The POLRMT inhibitor potently inhibited cell viability and reduced CCK-8 OD (Fig. 1I) in the primary and established endometrial carcinoma cells. Moreover, IMT1 (0.5 μM) inhibited cell proliferation (EdU incorporation, Fig. 1J) and migration (Fig. 1K). In primary human endometrial epithelial cells, “phEE-1” and “phEE-2”, treatment with IMT1 (0.5 μM) failed to significantly inhibit the viability and proliferation, as the CCK-8 OD (Fig. 1L) and EdU incorporation (Fig. 1M) were not significantly altered.

### IMT1 provokes apoptosis in human endometrial carcinoma cells

**Fig. 2:**
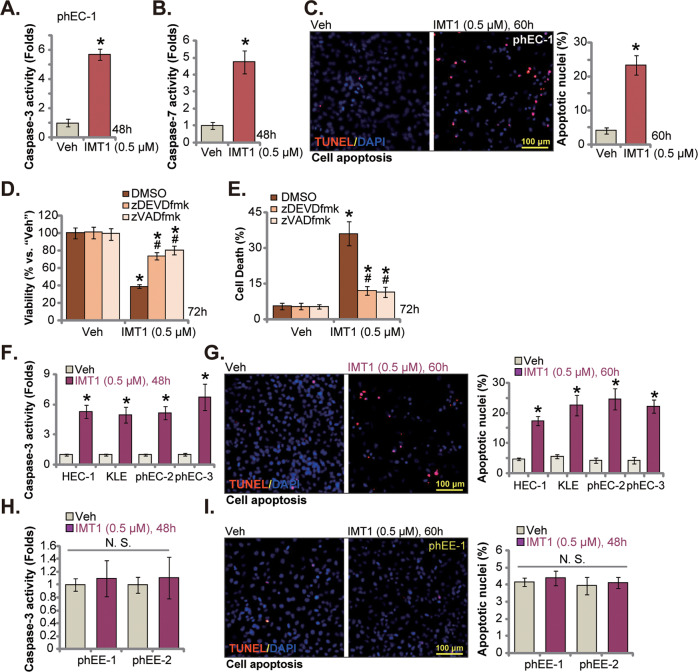
IMT1 provokes apoptosis in human endometrial carcinoma cells. The primary human endometrial carcinoma cells, phEC-1, were treated with IMT1 (0.5 μM) and were further cultivated for designated time, the caspase-3 activity and the caspase-7 activity were tested (**A** and **B**); Cell apoptosis was measured by nuclear TUNEL staining assays (**C**). The phEC-1 cells were pretreated for 1 h with 40 μM of z-DEVD-fmk, 40 μM of z-VAD-fmk or 0.1% DMSO (“DMSO”), followed by IMT1 (0.5 μM) treatment, and cells were further cultivated for additional 72 h, cell viability and death were tested separately by CCK-8 (**D**) and Trypan blue staining (**E**) assays. The immortalized endometrial carcinoma cell lines (HEC-1/KLE), the other primary cancer cells (“phEC-2/-3”), or the human endometrial epithelial cells (“phEE-1/-2”) were treated with IMT1 (0.5 μM), cells were further cultivated for designated hours, the caspase-3 activity (**F** and **H**) and cell apoptosis (by measuring nuclear TUNEL staining, **G** and **I**) were tested. “Veh” stands for the vehicle control (0.1% DMSO). Data were presented as mean ± standard deviation (SD, *n* = 5). *P < 0.05 vs. “Veh”. ^#^*P* < 0.05 vs. “DMSO” group (**D** and **E**). “N.S.” stands for the non-statistical difference (*P* > 0.05). The experiments were repeated five times with similar results obtained. Scale bar = 100 μm.

IMT1 treatment resulted in proliferation suppression, cell cycle arrest, and cell death, which together could cause cell apoptosis in endometrial carcinoma cells. Indeed, we found that the caspase-3 activity and the caspase-7 activity were boosted in IMT1 (0.5 μM)-treated phEC-1 primary cancer cells (Fig. 2A, B). Significant apoptosis was detected in IMT1-stimulated phEC-1 cells, as the TUNEL positively-stained nuclei (“apoptotic nuclei”) percentage was robustly increased (Fig. 2C). To test whether apoptosis was the primary reason of IMT1 (0.5 μM)-induced cytotoxicity in phEC-1 cells, the caspase-3 specific inhibitor z-DEVD-fmk and the pan caspase inhibitor z-VAD-fmk were utilized. We found that IMT1 (0.5 μM)-induced viability (CCK-8 OD) reduction (Fig. 2D) and cell death (Fig. 2E) were ameliorated by caspase inhibitors.

In the immortalized cell lines (HEC-1/KLE) and other primary endometrial carcinoma cells (“phEC-2/phEC-3”), treatment with IMT1 (0.5 μM) activated Caspase-3 (Fig. 2F) and increased nuclear TUNEL-positive staining (Fig. 2G), supporting apoptosis activation. In the primary human endometrial epithelial cells, “phEE-1” and “phEE-2”, the exact same IMT1 (0.5 μM) treatment failed to provoke significant apoptosis, as the Caspase-3 activity (Fig. 2H) and TUNEL-nuclei ratio (Fig. 2I) were not significantly increased. These results again supported the cancer cell specific effect by the POLRMT inhibitor.

### IMT1 impairs mitochondrial function in human endometrial carcinoma cells

**Fig. 3:**
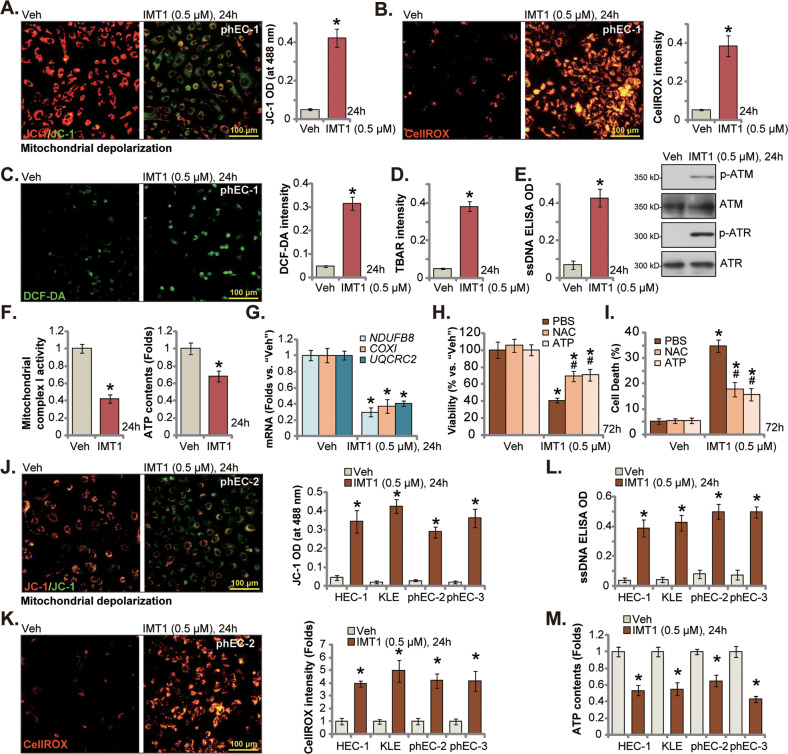
IMT1 impairs mitochondrial function in human endometrial carcinoma cells. The primary human endometrial carcinoma cells, phEC-1, were treated with IMT1 (0.5 μM) and were further cultivated for designated time, mitochondrial depolarization was tested by JC-1 fluorescence staining (**A**), and cellular ROS levels measured by CellROX and DCF-DA fluorescence staining (**B** and **C**); The lipid peroxidation was tested by the TBAR intensity assay (**D**) with DNA damages quantified via ssDNA ELISA (**E**); ATM-ATR phosphorylation and expression were tested as well (**E**). The mitochondrial complex I activity (**F**) and cellular ATP contents (**F**) were measured as well. *NDUFB8*, *COXI* and *UQCRC2* expression was tested via qRT-PCR assays (**G**). The phEC-1 cells were pretreated for 1 h with 400 μM of NAC, 2.5 mM ATP or PBS, followed by IMT1 (0.5 μM) treatment, and cells were further cultivated for additional 72 h, cell viability and death were tested separately by CCK-8 (**H**) and Trypan blue staining (**I**) assays. The immortalized endometrial carcinoma cell lines (HEC-1/KLE) or the other primary cancer cells (“phEC-2/phEC-3”) were treated with IMT1 (0.5 μM), cells were further cultivated for designated hours, mitochondrial depolarization and cellular ROS were tested by JC-1 (**J**) and CellROX fluorescence staining (**K**) assays respectively; The ssDNA contents (**L**) and ATP contents (**M**) were measured as well. “Veh” stands for the vehicle control (0.1% DMSO). Data were presented as mean ± standard deviation (SD, *n* = 5). * *P* < 0.05 vs. “Veh”. ^#^*P* < 0.05 vs. “PBS” group (**H** and **I**). The experiments were repeated five times with similar results obtained. Scale bar = 100 μm.

PLORMT is vital for mtDNA transcription, OXPHOS and biogenesis, PLORMT inhibition, silencing or KO should cause mitochondrial dysfunctions [14, 17, 24, 32]. Results in Fig. 3A revealed that IMT1 (0.5 μM) treatment in phEC-1 primary cancer cells induced mitochondrial depolarization and induced JC-1 red fluorescence conversion to green fluorescence monomers (Fig. 3A). Moreover, treatment with the POLRMT inhibitor induced robust ROS production, as the CellROX red fluorescence intensity (Fig. 3B) and the green DCF-DA fluorescence intensity (Fig. 3C) were both significantly boosted. IMT1 induced significant lipid peroxidation and increased TBAR activity in phEC-1 cells (Fig. 3D). Moreover, ROS production and oxidative injury were accompanied with robust DNA damage, which was evidenced by the increased ssDNA intensity and increased phosphorylation of ATM and ATR (Fig. 3E). Furthermore, the mitochondrial complex I activity (Fig. 3F) was inhibited and ATP levels were decreased (Fig. 3F) following treatment with IMT1 (0.5 μM) in phEC-1 cells. The number of mitochondrial transcripts, including *NDUFB8*, *COXI* and *UQCRC2*, was decreased in IMT1-treated phEC-1 cells (Fig. 3G). These results confirmed that IMT1 disrupted mitochondrial functions, leading to mitochondrial depolarization, ROS production, DNA damage as well as ATP depletion and mtDNA transcription inhibition in primary endometrial carcinoma cells.

Importantly, supplement with NAC or ATP inhibited IMT1 (0.5 μM)-induced cytotoxicity in phEC-1 cells (Fig. 3H, I), as NAC and ATP partially restored phEC-1 cell viability (Fig. 3H) and inhibited cell death (Fig. 3I). These results implied that IMT1-provoked mitochondrial dysfunction should be one main mechanism of IMT1-induced cytotoxicity in endometrial carcinoma cells. Mitochondrial dysfunctions were also detected in IMT1-treated immortalized endometrial carcinoma cell lines (HEC-1/KLE) and other primary cells (“phEC-2/phEC-3”). As shown, treatment with the POLRMT inhibitor induced mitochondrial depolarization (JC-1 green monomers accumulation, Fig. 3J) and ROS production (Fig. 3K). DNA damage, or ssDNA increasing (Fig. 3L), and ATP depletion (Fig. 3M) were observed as well in the endometrial carcinoma cells after IMT1 treatment.

### POLRMT depletion impairs mitochondrial functions and induces robust anti-cancer cell activity in human endometrial carcinoma cells

**Fig. 4:**
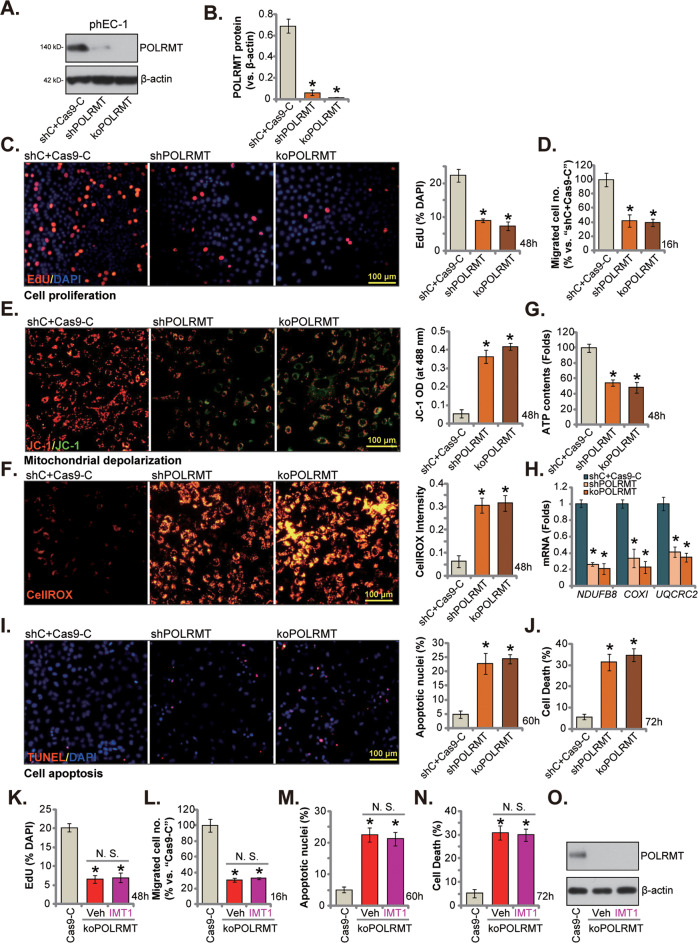
POLRMT depletion impairs mitochondrial functions and induces robust anti-cancer cell activity in cultured endometrial carcinoma cells. The primary human endometrial carcinoma cells, phEC-1, expressing the lentiviral POLRMT shRNA (“shPOLRMT”), the lentiviral CRISPR/Cas9-POLRMT-KO construct (“koPOLRMT”), or the lentiviral scramble control shRNA plus Cas9 control construct (“shC+Cas9-C”), were established, and POLRMT protein expression detected (**A** and **B**). Cells were further cultivated for indicated time periods, EdU incorporation (**C**) and in vitro cell migration (**D**) were measured. Mitochondrial depolarization was tested by JC-1 fluorescence staining (**E**), and cellular ROS levels measured by CellROX staining (**F**); The cellular ATP contents were measured as well (**G**) and *NDUFB8*, *COXI* and *UQCRC2* expression tested via qRT-PCR assays (**H**). Cell apoptosis (**I**) and death (**J**) were tested by nuclear TUNEL staining and Trypan blue staining assays, respectively. The single stable koPOLRMT phEC-1 cells were treated with IMT1 (0.5 μM) and were further cultivated for designated time, EdU incorporation (**K**) and in vitro cell migration (**L**) were measured. Cell apoptosis (**M**) and death (**N**) were tested by nuclear TUNEL staining and Trypan blue staining assays, respectively. POLRMT protein expression was shown (**O**). Data were presented as mean ± standard deviation (SD, n = 5). *P < 0.05 vs. “shC+Cas9-C”/“Cas9-C” cells. “N.S.” stands for the non-statistical difference (*P* > 0.05). The experiments were repeated five times with similar results obtained. Scale bar = 100 μm.

Since POLRMT inhibition by IMT1 induced robust anti-endometrial carcinoma cell activity, we further hypothesized that POLRMT depletion should exert the similar actions. To this purpose, POLRMT shRNA-containing lentivirus was added to phEC-1 cancer cells, stable “shPOLRMT” cells were formed after puromycin selection (see Methods). Alternatively, a lentiviral CRISPR/Cas9-POLRMT-KO construct [24, 32] was utilized to knockout POLRMT in phEC-1 cancer cells (“koPOLRMT” cells) (see “Methods”). Control cells were treated with the lentiviral scramble control shRNA plus CRISPR/Cas9-control construct (“shC+Cas9-C”). As shown, PLORMT protein expression was robustly decreased in shPOLRMT and koPOLRMT phEC-1 cells (Fig. 4A, B). Importantly, PLORMT shRNA or KO potently inhibited phEC-1 cell proliferation and decreased EdU-positively stained nuclei ratio (Fig. 4C). The in vitro cell migration (Fig. 4D) was largely inhibited in POLRMT-silenced/-KO phEC-1 cells.

PLORMT depletion impaired mitochondrial functions in phEC-1 cells and induced mitochondrial depolarization (Fig. 4E). The latter was evidenced by accumulation of JC-1 green monomers in shPOLRMT and koPOLRMT phEC-1 cells (Fig. 4E). Moreover, ROS production and oxidative injury were detected in phEC-1 cells with PLORMT depletion. The CellROX red fluorescence intensity (Fig. 4F) was substantially increased in phEC-1 cells with POLRMT silencing or KO. The cellular ATP contents were however decreased in POLRMT-depleted cells (Fig. 4G), where the number of mitochondrial transcripts, including *NDUFB8*, *COXI* and *UQCRC2*, were substantially decreased (Fig. 4H). In addition, increased nuclear TUNEL staining supported that PLORMT shRNA or KO induced apoptosis activation in phEC-1 cancer cells (Fig. 4I). Moreover, PLORMT depletion caused phEC-1 cell death and increased Trypan blue-positive staining cells (Fig. 4J). Therefore, POLRMT shRNA/KO impaired mitochondrial functions and induced robust anti-cancer cell activity in phEC-1 cancer cells.

Importantly, IMT1 was ineffective in koPOLRMT phEC-1 cells. POLRMT KO-induced proliferation inhibition (Fig. 4K), migration reduction (Fig. 4L), cell apoptosis (Fig. 4M) and death (Fig. 4N) were not further intensified by co-treatment of IMT1 (0.5 μM). POLRMT protein expression was again null in koPOLRMT phEC-1 cells with or without IMT1 treatment (Fig. 4O). These results supported that POLRMT is the primary target of IMT1 in endometrial carcinoma cells.

### Ectopic overexpression of POLRMT exerts cancer-promoting activity in endometrial carcinoma cells

**Fig. 5:**
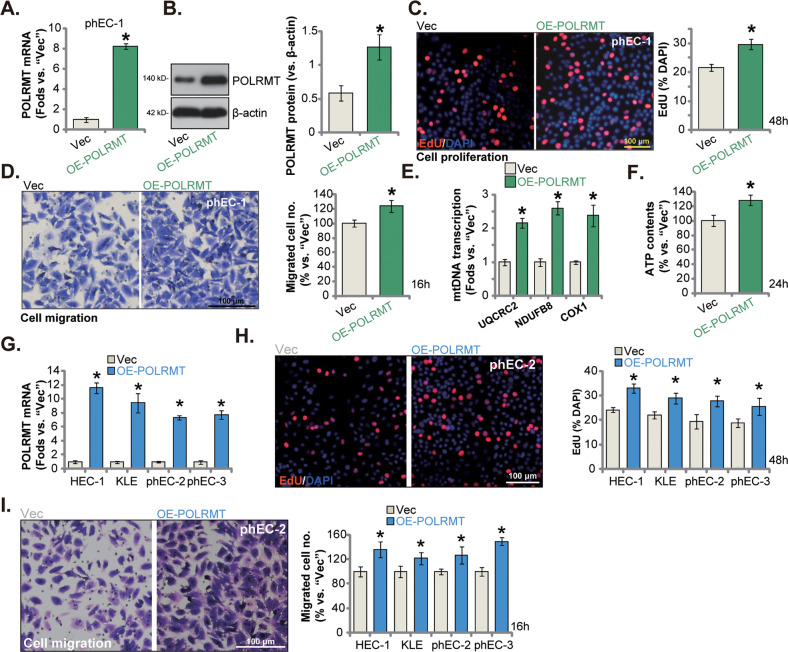
Ectopic overexpression of POLRMT exerts cancer-promoting activity in endometrial carcinoma cells. The primary human endometrial carcinoma cells (“phEC-1/-2/-3”) or the immortalized cell lines (HEC-1/KLE), expressing the lentiviral POLRMT-overexpressing construct (“OE-POLRMT”) or the empty vector (“Vec”), were formed. POLRMT *mRNA* and protein expression detected (**A**, **B** and **G**). Cells were further cultivated for indicated time periods, EdU incorporation (**C** and **H**), in vitro cell migration (**D** and **I**) were measured. Expression of mtDNA transcripts (**E**) and cellular ATP contents were measured as well (**F**). Data were presented as mean ± standard deviation (SD, *n* = 5). * ***P*** < 0.05 vs. “Vec” cells. The experiments were repeated five times with similar results obtained. Scale bar = 100 μm.

Ectopic overexpression of POLRMT should exert opposite activity and cancer-promoting functions. To verify this hypothesis, the lentivirus-packed POLRMT-overexpressing construct was transduced to the phEC-1 primary cancer cells, and with selection by puromycin stable cells formed. These cells were named as “OE-POLRMT” cells. Comparing to the vector control cells (“Vec”), expression levels of *POLRMT* mRNA (Fig. 5A) and protein (Fig. 5B) were both dramatically elevated in the OE-POLRMT phEC-1 cells. Overexpression of POLRMT enhanced phEC-1 cell proliferation and increased nuclear EdU incorporation (Fig. 5C). The phEC-1 cell in vitro migration (Fig. 5D) was accelerated following POLRMT overexpression. Moreover, mtDNA transcription was augmented in the OE-POLRMT phEC-1 cells and *NDUFB8*, *UQCRC2* and *COXI* transcripts were significantly increased (Fig. 5E). In addition, the cellular ATP contents were increased in POLRMT-overexpressed cells (Fig. 5F).

The POLRMT-overexpressing lentivirus was also added to the immortalized cell lines (HEC-1/KLE) or other primary endometrial carcinoma cells (“phEC-2/phEC-3”). Following selection POLRMT-overexpressing cells, “OE-POLRMT”, were formed, showing increased *POLRMT* mRNA expression (Fig. 5G). Ectopic overexpression of POLRMT augmented cell proliferation (EdU assays, Fig. 5H) and in vitro cell migration (Fig. 5I) in the immortalized and primary endometrial carcinoma. These results showed that POLRMT overexpression exerted cancer-promoting activity in endometrial carcinoma cells.

### Oral administration of IMT1 hinders the growth of endometrial carcinoma xenograft in nude mice

**Fig. 6:**
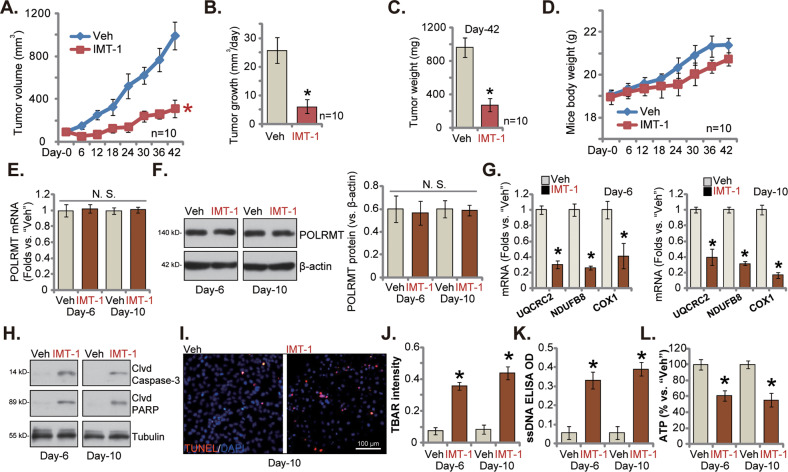
Oral administration of IMT1 hinders the growth of endometrial carcinoma xenograft in nude mice. The phEC-1 xenograft-bearing nude mice were orally-administrated with IMT1 (50 mg/kg body, every 48 h) or the vehicle control (“Veh”), the phEC-1 xenograft volumes (**A**) and animal body weights (**D**) were recorded every 6 days. The estimated daily tumor growth, in mm^3^ per day, was calculated (**B**). All mice were then sacrificed by decapitation at “Day-42”, and xenograft tumors were isolated and weighed (**C**). At Day-6 and Day-10, 12 h after IMT1/ vehicle administration, one phEC-1 xenograft from each group was isolated, expression of listed genes and proteins in the described phEC-1 xenograft tissues was shown (**E**–**H**); The TBAR intensity (**J**), ssDNA contents (**K**) and ATP levels (**L**) were also measured. Alternatively, the phEC-1 xenograft slides were subject to immunofluorescence staining of TUNEL-positive nuclei (**I**). Data were mean ± standard deviation (SD). *P < 0.05 versus “Veh” group. Scale bar = 100 μm.

At last we tested the anti-cancer activity of IMT1 in vivo. The primary phEC-1 cells, at six million cells per mouse, were injected subcutaneously (s.c.) to the flanks of the nude mice. The subcutaneous phEC-1 xenografts were formed after 18 days, and each xenograft close to 100 mm^3^. It was labeled as “Day-0”. The phEC-1 xenograft-bearing mice were then orally administrated with IMT1 or vehicle control [17]. IMT1 was administrated at 50 mg/kg body weights, every 48 h for five rounds (at day-0, day-2, day-4, day-6 and day-8). The tumor growth curve results showed that IMT1 administration robustly inhibited phEC-1 xenograft growth in the nude mice (Fig. 6A). The estimated daily phEC-1 xenograft growth, in mm^3^ per day, was substantially decreased with IMT1 administration (Fig. 6B). At Day-42, all phEC-1 xenografts of the IMT1 group and the vehicle control group were isolated carefully and were weighted. IMT1-administrated phEC-1 xenografts were significantly lighter than vehicle control-treated tumors (Fig. 6C). The mice body weights, on the other hand, were not significantly different between IMT1-administrated or vehicle-treated mice (Fig. 6D). We also failed to observe apparent toxicities in the IMT1-treated mice, which was in line with previous findings [17]. Thus, the growth of phEC-1 xenografts in nude mice was largely inhibited after IMT1 administration.

Signaling changes in the xenograft tissues were analyzed. Specifically, at day-6 and day-10, 12 h after IMT1/vehicle administration, one phEC-1 xenograft from each group was isolated. The xenograft tissue lysates of the four isolated tumors were analyzed. As demonstrated, IMT1 did not alter *POLRMT* mRNA (Fig. 6E) and protein (Fig. 6F) expression in phEC-1 xenograft tissues. However, the number of POLRMT-dependent mitochondrial transcripts, including *NDUFB8*, *UQCRC2* and *COXI*, was significantly decreased in IMT1-treated phEC-1 xenograft tissues (Fig. 6G). Contrarily, cleaved-caspase-3 and cleaved-PARP levels were significantly elevated (Fig. 6H). Further supporting apoptosis induction, the tissue immunofluorescence staining assay results showed that the nuclear TUNEL staining was largely increased in IMT1-administrated tumor slides (Fig. 6I). When compared to the vehicle-treated control xenografts, we found that the TBAR intensity (Fig. 6J) and ssDNA contents (Fig. 6K) were robustly elevated in phEC-1 xenograft tissues with IMT1 treatment. Whereas the ATP contents were decreased in IMT1-administrated phEC-1 xenograft tissues (Fig. 6L). These results supported that mitochondrial functions in phEC-1 xenograft tissues were impaired after IMT1 administration.

## Discussion

Currently, the application of sentinel lymph node biopsy and/or minimally invasive surgical staging have increased the efficiency of resection surgery for endometrial carcinoma patients [3, 33–36]. Moreover, postoperative radiotherapy could decrease local or regional metastasis of the cancer in intermediate/high-risk patients. Adjuvant targeted agents, including PD-1/PD-L1 blockers, are being utilized in recurrent and other endometrial carcinoma patients [3, 33–36]. Yet, for many advanced patients the prognosis is far from satisfactory [3, 33–36]. Thus, it is important to explore novel molecularly-targeted agents against the devastating disease [37].

Recent findings have suggested that certain cancers heavily rely on OXPHOS and therefore OXPHOS inhibition is effective in inhibiting growth of these cancer cells [6, 9]. A meta-analysis of 31 different cancer cell lines and 16 normal cell lines have demonstrated that the respective contribution of OXPHOS and glycolysis to production of ATP is highly variable among different cell types, and the average OXPHOS contribution to ATP production is 80% in normal cells and is strikingly 83% in cancer cells [38]. Indeed, O_2_ availability in solid cancer is the primary factor to determine of the oxygen consumption rate, supporting that mitochondrial respiratory capacity is not likely functionally disabled in cancer cells [39].

Previous studies have revealed that POLRMT could be a novel and key oncogenic gene in several cancers. Its overexpression is important for OXPHOS, mitochondrial biogenesis and the growth of different cancer cells. Zhou et al., have shown that POLRMT expression is significantly elevated in non-small cell lung cancer (NSCLC) tissues and cells, required for NSCLC cell growth in vitro and in vivo. Conversely, POLRMT silencing or KO disrupted mitochondrial functions, impaired ATP production and suppressed NSCLC proliferation, migration, and invasion [24]. Han et al., discovered that *POLRMT* mRNA and protein expression is upregulated in osteosarcoma tissues and cells [32]. Whereas POLRMT depletion hindered osteosarcoma cell proliferation and migration in vitro, and inhibited osteosarcoma xenograft growth in nude mice [32]. Wang et al., also revealed POLRMT overexpression in skin squamous cell carcinoma (SCC) tissues and cells, essential for mitochondrial function and skin SCC cell growth [19].

Here the results of the present study supported that POLRMT could be an important therapeutic target of endometrial carcinoma. In both primary endometrial carcinoma cells and established cell lines, IMT1 potently inhibited viability, proliferation, cell-cycle progression, and motility, while inducing caspase-apoptosis activation. Genetic evidences supported that shRNA-induced silencing or CRISPR/Cas9-caused KO of POLRMT similarly induced robust anti-cancer cell activity in cultured endometrial carcinoma cells. Importantly, IMT1 failed to induce further cytotoxicity in POLRMT-KO endometrial carcinoma cells. Conversely, ectopic overexpression of POLRMT, by the lentiviral construct, further augmented endometrial carcinoma cell proliferation and motility. In vivo, oral administration of a single dose of IMT1 robustly inhibited endometrial carcinoma xenograft growth in the nude mice. Importantly, IMT1 failed to induce significant cytotoxicity in primary human endometrial epithelial cells. In vivo, oral administration of IMT1 also failed to induce apparent toxicities in nude mice. Thus, targeting POLRMT by IMT1 potently inhibited endometrial carcinoma cell growth in vitro and in vivo. It is likely that POLRMT inhibition/silencing only targeted the enhanced mitochondrial functions (including OXPHOS) in endometrial carcinoma cells, this explains the ineffectiveness of IMT-1 treatment/POLRMT silencing in primary endometrial epithelial cells.

Endometrial carcinoma, like several other human cancers, have increased levels of mtDNA [6, 7, 9, 39–41]. Moreover, endometrial carcinoma with high proliferative and metastatic potential rely heavily on OXPHOS for ATP production than normal epithelial cells [6, 7, 9, 39]. Thus targeting OXPHOS could result in profound mitochondrial dysfunction and energy crisis, thereby inhibiting cancer cell growth [6, 7, 9, 39–41]. POLRMT is vital for mtDNA transcription, OXPHOS and mitochondrial biogenesis [16, 17, 42]. Here we found that treatment with the PLORMT inhibitor IMT1 impaired mitochondrial functions, causing mtDNA transcription inhibition, mitochondrial mitochondrial depolarization, ROS production, and more importantly mitochondrial complex I activity inhibition and ATP reduction in endometrial carcinoma cells. Moreover, mtDNA transcription inhibition, oxidative stress and ATP loss were also detected in IMT1-treated endometrial carcinoma xenograft tissues. mtDNA transcription and ATP contents were however increased in POLRMT-overexpressed endometrial carcinoma cells. The antioxidant NAC or ATP supplement alleviated IMT1-induced cytotoxicity in endometrial carcinoma cells. Therefore, mitochondrial dysfunction and energy crisis should be the primary mechanism of IMT1-induced anti-endometrial carcinoma activity.

## Conclusion

Exploring novel therapies for endometrial carcinoma is important. [1, 2]. In depth understanding the pathological mechanisms of endometrial carcinoma progression should advance molecularly-targeted strategies [3–5]. The results of this study show that PLORMT inhibition by IMT1 efficiently suppresses endometrial carcinoma cell growth in vitro and in vivo. IMT1, alone or as a combinatory therapy, could be further evaluated as a potential anti-endometrial carcinoma agent.

## Supplementary information

Authorship_change_form

SUPPLEMENTAL Figure 1

aj-checklist form

Author contribution form

## Data Availability

All data are available upon request.
