# Supplementary figures and images for: A first-in-class POLRMT specific inhibitor IMT1 suppresses endometrial carcinoma cell growth
Source: Cell Death Dis. 2023 Feb 23;14(2):152. doi: 10.1038/s41419-023-05682-7 (PMC9950144; doi:10.1038/s41419-023-05682-7)

Figure S1. The uncropped blotting images of the study.

Figure 3

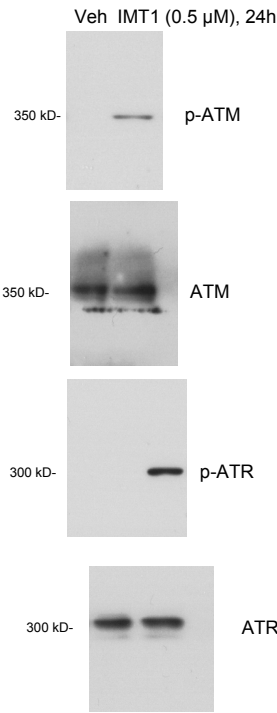

Figure 4

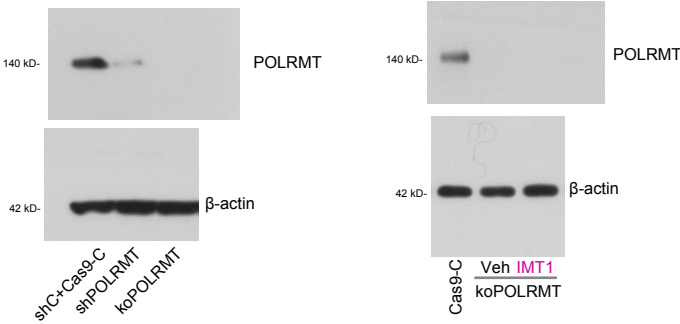

Figure 5

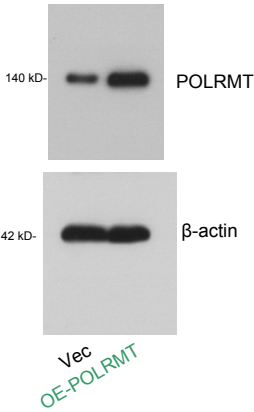

Figure 6.

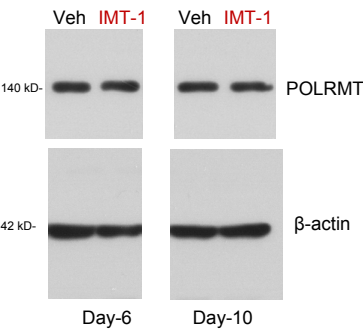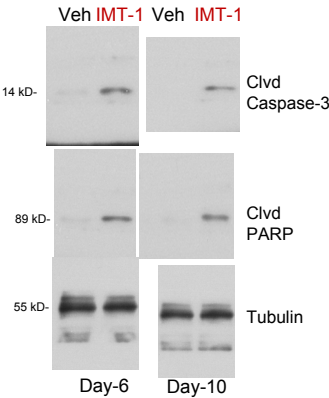

Supplement: Supplementary file 2 — SUPPLEMENTAL Figure 1 [file 41419_2023_5682_MOESM2_ESM.pdf]
