# Supplementary material for: A first-in-class POLRMT specific inhibitor IMT1 suppresses endometrial carcinoma cell growth
Source: Cell Death Dis. 2023 Feb 23;14(2):152. doi: 10.1038/s41419-023-05682-7 (PMC9950144; doi:10.1038/s41419-023-05682-7)
Supplement: Supplementary file 4 — Author contribution form [file 41419_2023_5682_MOESM4_ESM.pdf]

DECLARATION OF CONTRIBUTIONS TO ARTICLE

ADMC

Manuscript Number: CDDIS-22-5315R

Journal Name: *Cell Death & Disease* (the 'Journal')

Proposed Title of the Contribution:

A first-in-class POLRMT specific inhibitor IMT1 suppresses endometrial carcinoma cell growth (the 'Contribution)

Author(s):

Shu-ping Li, Li Ou, Yan Zhang,Fang-rong Shen and You-guo Chen (the 'Authors')

For all *CDDis* articles, each person named as an author in the published version must be able to show he or she has contributed substantially to the article.

Authorship credit should be based on 1) substantial contributions to conception and design, acquisition of data, or analysis and interpretation of data; 2) drafting the article or revising it critically for important intellectual content; and 3) final approval of the version to be published. Authors should meet conditions 1, 2 and 3.

Any person who cannot be shown to have made a substantial contribution to the article cannot be listed as an author in the final version. The name of any person who is deemed to have made a minor contribution can, however, appear in the Acknowledgments section of the article.

Please complete the table below to indicate the contributions of all named authors to the manuscript.

Author Full Name:	Specification of Contribution to the Manuscript:
Shu-ping Li	Culture primary endometrial carcinoma cells and normal epithelial cells, testing testing signalings, performed the in vivo studies, drafting the article and revising it, with final approval of the version submitted to the journal.
Li Ou	Culture primary endometrial carcinoma cells and normal epithelial cells, testing testing signalings, performed the in vivo studies, drafting the article and revising it, with final approval of the version submitted to the journal.
Yan Zhang	Culture primary endometrial carcinoma cells and normal epithelial cells, testing testing signalings, performed the in vivo studies, drafting the article and revising it, with final approval of the version submitted to the journal.
Fang-rong Shen	Culture primary endometrial carcinoma cells and normal epithelial cells, testing testing signalings, performed the in vivo studies, drafting the article and revising it, with final approval of the version submitted to the journal.
You-guo Chen	Culture primary endometrial carcinoma cells and normal epithelial cells, testing testing signalings, performed the in vivo studies, drafting the article and revising it, with final approval of the version submitted to the journal.

Please complete the table below to indicate the contributions of all named authors to the figures.

Figure 1:

SL, LO, YZ FS, and YC

Figure 2:

SL, LO, YZ FS, and YC

Figure 3:

SL, LO, YZ FS, and YC

Figure 4:

SL, LO, YZ FS, and YC

Figure 5:

SL, LO, YZ FS, and YC

Figure 6:

SL, LO, YZ FS, and YC

Signed for and on behalf of the Author(s):	Print Name:	Date:
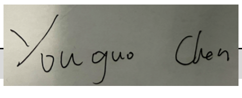	You-guo Chen	02/02/2023
